# Effects of One-Step Laparoscopic Abomasopexy and Laparotomic Omentopexy Treatments of Left Displaced Abomasum on Metabolic and Inflammatory Stress Responses, Liver Health and Milk Yield in Multiparous Cows

**DOI:** 10.3390/ani16213366

**Published:** 2026-10-25

**Authors:** Sveta Arsić, Radiša Prodanović, Sreten Nedić, Dušan Bošnjaković, Ivan Vujanac, Danijela Kirovski, Predrag Simeunović, Aleksandra Mitrović, Ljubomir Jovanović, Jovan Bojkovski, Sunčica Borozan, Svetlana Nedić

**Affiliations:** 1 Department of Ruminant and Swine Diseases, Faculty of Veterinary Medicine, University of Belgrade, Bulevar Oslobodjenja 18, 11000 Belgrade, Serbia; sarsic@vet.bg.ac.rs (S.A.); prodanovic@vet.bg.ac.rs (R.P.); sreten.nedic@vet.bg.ac.rs (S.N.); vujanac@vet.bg.ac.rs (I.V.); aleksandra.mitrovic@vet.bg.ac.rs (A.M.); jovan_bojkovski@yahoo.com (J.B.); 2 Department of Physiology and Biochemistry, Faculty of Veterinary Medicine, University of Belgrade, Bulevar Oslobodjenja 18, 11000 Belgrade, Serbia; dusan.bosnjakovic@vet.bg.ac.rs (D.B.); dani@vet.bg.ac.rs (D.K.); ljubomir.jovanovic@vet.bg.ac.rs (L.J.); 3 Department of General Education, Faculty of Veterinary Medicine, University of Belgrade, Bulevar Oslobodjenja 18, 11000 Belgrade, Serbia; suncicaborozan58@gmail.com; 4 Department of Reproduction, Fertility and Artificial Insemination, Faculty of Veterinary Medicine, University of Belgrade, Bulevar Oslobodjenja 18, 11000 Belgrade, Serbia; svetlana.nedic@vet.bg.ac.rs

**Keywords:** multiparous cows, abomasum, laparoscopic abomasopexy, laparotomic omentopexy, left displaced abomasum, myeloperoxidase, oxidative stress, dairy cow

## Abstract

Metabolic and inflammatory stress during the postoperative period following left displaced abomasum (LDA) correction poses significant risks to dairy cows’ health and productivity. This study aimed to evaluate the differences in metabolic and inflammatory responses between LDA cows treated with laparoscopic abomasopexy in one-step and left paralumbar fossa omentopexy, focusing on myeloperoxidase (MPO), metabolic- and liver-related biochemical markers, antioxidative response, and milk yield. Sixteen LDA cows were randomly assigned to one-step laparoscopic abomasopexy (LPS, *n* = 8) or left laparotomic omentopexy (LPT, *n* = 8), matched by parity and days in milk with healthy cows (CON, *n* = 8), and evaluated for blood indices and milk production during lactation. Blood samples were collected before (D0) surgery and 1 (D1), 3 (D3), 10 (D10), and 30 (D30) days following surgery, while milk yield was recorded monthly. MPO activity exhibited significantly higher values in both LPS (*p* < 0.001) and LPT (*p* < 0.001) cows compared to CON on D1, but also significantly lower values in LPS than in LPT cows (*p* < 0.001). In addition, both LPS and CON cows displayed significantly lower MPO levels than LPT cows on D3 (*p* < 0.001; *p* < 0.001; respectively), D10 (*p* < 0.001; *p* < 0.001; respectively), and D30 (*p* < 0.001; *p* = 0.008; respectively). Serum paraoxonase-1 (PON1) activity was significantly suppressed on D1 in both the LPS and LPT groups compared to CON cows (*p* < 0.05); however, overall PON1 activity remained significantly lower in the LPT group throughout the monitoring period (*p* = 0.016), despite a non-significant treatment × day interaction (*p* = 0.099). Significant treatment × day interactions were found for total bilirubin concentrations (*p* = 0.019), aspartate aminotransferase (AST, *p* < 0.001), and gamma-glutamyl transferase (GGT, *p* = 0.011) activities. Compared to control, AST and GGT activities were significantly elevated on D1 in both LPS (*p* = 0.037; *p* = 0.028, respectively) and LPT (*p* = 0.007; *p* = 0.045, respectively), while total bilirubin concentrations showed marked elevation on D1 in the LPT group compared to both CON and LPS cows (*p* = 0.031). Non-esterified fatty acids, beta-hydroxybutyrate and total bilirubin showed more favorable patterns in the LPS compared to LPT group too. Compared to LPT cows, milk yield was higher in LPS cows on days 30 (*p* < 0.001), 60 (*p* = 0.016), and 90 (*p* = 0.005) of lactation. In conclusion, this study underlines the benefits of laparoscopic abomasopexy in one step during the postoperative period, as it reduced the inflammatory response, could positively impact recovery of milk production, and eventually enhanced liver metabolism in LDA cows. In addition, our results suggest that MPO shows potential for use as an exploratory biomarker for monitoring the scale of postoperative tissue damage in dairy cows.

## Simple Summary

Left displaced abomasum (LDA) is a common digestive disorder of high-producing dairy, cows typically occurring in the first 6 weeks of lactation, which often leads to reduced health and performance as well as an increased risk of culling. Selection of proper surgical technique for the reposition of displaced abomasum may significantly impact the speed and quality of cow recovery, thereby minimizing the negative consequences on milk production and reproductive success. Among the various blood biomarkers used to evaluate health status of LDA cows, the results of this trial showed that myeloperoxidase (MPO) shows potential as an exploratory biomarker of pre- and especially post-surgical oxidative stress and inflammation. The one-step laparoscopic abomasopexy used in this study for LDA correction exhibited advantages over the Utrecht method by reducing postoperative inflammation and liver function disruption while contributing to a satisfactory 305-day milk production.

## 1. Introduction

Abomasal displacements are considered one of the most important digestive disorders of dairy cows, causing adverse outcomes on health and productivity [1]. During the course of this disease, the abomasum can be shifted to the left side of the abdomen between the rumen and the abdominal wall (left displacement of abomasum, LDA) or to the right caudo-dorsal position (right displacement of abomasum, RDA), with the potential to twist on the lesser omentum, creating an abomasal volvulus (AV) [2]. The occurrence of LDA in dairy cows ranged from 4 to 4.7 times higher than RDA [3] with 85% to 91% of LDA cases diagnosed in the first 6 weeks after calving [4]. Besides significant milk production losses, high treatment costs and an increased risk of culling are the primary causes of the financial losses associated with this disease [5].

The development of LDA in dairy cows has been linked to several factors, including breed, age, parity, genetics, nutrition and energy metabolism, season, and other periparturient events [6]. However, abomasal hypomotility and atony with the subsequent accumulation of gases and fluid constitute the pivotal event triggering the development of this disorder [7,8]. Among a variety of interrelated factors, decreased abomasal motility has been associated with elevated peripartum blood levels of non-esterified fatty acids (NEFA) and β-hydroxybutyrate (BHB), indicating the extent of negative energy balance (NEB) encountered by dairy cows during the transition period [1]. Furthermore, the increased mobilization of lipids during NEB stimulates the production of reactive oxygen species (ROS), which in turn promotes the release of pro-inflammatory cytokines and acute phase proteins [9]. These metabolic and inflammatory responses may contribute to decreased abomasal motility and thus further to the pathogenesis of LDA [10].

The treatment of LDA can be either conservative (casting and rolling the cow) or surgical. There are several surgical approaches (which differ in their invasiveness) available for the treatment of LDA, and each has its advantages and disadvantages [11]. Right paralumbar fossa omentopexy or the Hannover approach is still considered a technique of choice in the UK, Germany, USA, Japan, and Canada due to the fact that several variations of a displaced abomasum can be corrected (LDA, RDA, AV) [11,12]. Left paralumbar fossa omentopexy or the Utrecht approach [8] is chosen by some practitioners for the benefits of direct visualization, especially where adhesions or peritonitis are suspected, enabling surgeons to inspect the abomasum before securing it [10]. Right paramedian abomasopexy, although widely used for LDA correction, is usually not selected as the first option for right abomasal disorders [10] due to issues in the localization and manipulation of the viscera caused by the position in dorsal recumbency. Percutaneous fixation with a toggle bar suture was developed as a cost-effective and rapid field technique for correcting LDA [13]; however, due to the fact that it is a closed, partially “blind” procedure, it carries notable postoperative clinical risks, such as peritonitis, abomasal fistulation and frequent recurrence of the condition [14]. Laparoscopic abomasopexy performed as a two-step [15] or one-step method [16,17] is a relatively fast, minimally invasive technique enabling direct visualization of the displaced abomasum along with the therapeutic safety and success rates of laparotomy [18]. In addition, laparoscopic treatment for resolving LDA, compared with classical surgical techniques, results in faster recovery and less disturbed milk production [15,19,20].

Stress significantly impacts cattle welfare and their capability to cope with ongoing health disorders. Previous studies in LDA cows have shown that affected cows experience more pronounced metabolic and inflammatory stress than their healthy counterparts [21,22]. Therefore, the implementation of appropriate treatment options can considerably reduce post-surgical stress and boost the recovery of treated cows, reducing milk losses and health complications [23,24]. A large body of literature has provided comparative data on the outcomes following different LDA surgical procedures, focusing on survival rates and production results [19,23]. In addition, the postoperative recovery success of different surgical approaches for managing LDA can be assessed through the analysis of hematological and biochemical biomarkers and acute-phase response [25]. Biomarkers of oxidative stress and inflammation, such as paraoxonase-1 (PON1) and myeloperoxidase (MPO), as well as the overall ability of the animal to withstand oxidative challenges, reflected in the total antioxidant capacity (TAC), can serve as valuable pre- and postoperative prognostic tools in cattle [22,26,27,28]. Even though several studies reported variations in the levels of some oxidative stress indices in cows before [22,29] and after LDA surgery [27,28,30], to the best of our knowledge, no published data describe the activity of MPO in LDA-treated cows.

In our previous study [8], we compared the effects of one-step laparoscopic abomasopexy with left paralumbar omentopexy on the post-surgical convalescence of primiparous cows evaluated through hematological and electrolyte parameters, concentrations of lactate and cortisol, and the influence of the performed surgical method on milk production and service period duration. Briefly, the results of the above-mentioned study suggested faster reestablishment of abomasal flow and a lower postoperative stress response, followed by improvements in milk production and reproductive parameters in laparoscopic-treated compared to left paralumbar omentopexy-treated primiparous cows. With that in mind, our current hypothesis posits that one-step laparoscopic abomasopexy exerts beneficial effects, thus reducing metabolic and inflammatory stress responses and facilitating milk production recovery during the postoperative period in multiparous cows, too. In addition, we hypothesized that the production response to the laparoscopic abomasopexy procedure could be mediated through reduced tissue damage and improved liver health. Therefore, the objective of this study was to compare metabolic parameters, oxidative stress biomarkers, and liver enzymes in healthy cows versus LDA cows (treated via laparoscopic and Utrecht approaches) and to evaluate the pattern of changes in these indices during the postoperative period in addition to lactation performance.

## 2. Materials and Methods

### 2.1. Experimental Site and Ethics Statement

This experiment was carried out concurrently with the trial on primiparous cows reported by [8]. The experimental protocol was approved by the Ethical Committee of the Faculty of Veterinary Medicine, University of Belgrade, in accordance with the National Regulations on Animal Welfare (No. 26/2020, from 23 September 2020), and the Veterinary Directorate under the Ministry of Agriculture, Forestry and Water Management of the Republic of Serbia (No. 323-07-11720/2020-05/5). The present study was conducted on a 1300-cow commercial dairy farm (AlDahra, Serbia, d.o.o.) near Belgrade, Serbia, at latitude 44°56′08.6″ North and longitude 20°28′44.5″ East.

### 2.2. Animals and Management

Following parturition, all cows on the selected farm were maintained in a fresh cow group with a tie-stall housing system for approximately 25 days, under controlled nutritional management and enhanced health monitoring by the veterinarians and herd staff. Cows were fed total mixed ration (TMR) delivered in two equal portions at 6:30 AM and 6:30 PM. The diet was formulated to meet or exceed the National Research Council Requirements for Dairy Cattle [31], with the ingredients and chemical composition of the early lactation ration shown in Table 1. Data concerning the ingredients list, forage analyses and ration composition were provided by a farm head nutritionist. The average dry matter intake (DMI) of the fresh group within this study was calculated on 24.0 kg/day. Cows were milked twice daily, at 6 AM and 6 PM.

### 2.3. Animals Selection and Experimental Groups

The study encompassed 24 multiparous Holstein cows selected from the group of fresh cows. During the morning evaluation of feed refusals, each cow consuming less than 80% of the delivered TMR—irrespective of a concurrent decline in milk yield—was examined for the presence of DA by a skilled veterinarian. The diagnosis of left displaced abomasum (LDA) was established based on clinical examination, characterized by the presence of a distinct ‘ping’ sound during auscultation combined with percussion and succussion of the left thoraco-abdominal wall.

Cows diagnosed with LDA—but presenting a body temperature ≤ 39 °C and showing no clinical signs of mastitis, metritis, or lameness—were systematically allocated to one of two experimental groups in an alternating sequence (1:1 ratio). The first group (LPS, *n* = 8) underwent one-step laparoscopic abomasopexy according to the method described by Christiansen [17]. The second group (LPT, *n* = 8) was treated via left paralumbar fossa omentopexy (the Utrecht approach), as previously described by Arsić et al. [8]. Additionally, eight clinically healthy multiparous cows, matched by parity, pre-LDA milk production and days in milk (DIM), were enrolled as a control group (CON, *n* = 8). To maintain balanced group assignment, one healthy control cow was selected following the sequential allocation of every two LDA cows into the experimental groups.

Cows included in the study were in their 3rd lactation, with a mean age of 4.59 ± 0.42, 4.89 ± 0.63, and 4.70 ± 0.36 years for the CON, LPS, and LPT groups, respectively. At the time of LDA occurrence, the average DIM of cows in the LPS and the LPT group was 13.86 ± 3.56 and 16.57 ± 2.92, respectively, whereas the average DIM of their selected matched control herd-mates was 15.70 ± 2.84. The overall prevalence of abomasal displacement on this farm was 8.44%, with an occurrence rate of 81.2% for LDA. There were no significant differences in age, number of previous lactations or DIM between the groups studied. Recurrence of LDA, as well as post-surgical complications, were not reported in cows in both LPT and LPS groups. All animals included in the study survived beyond 305-day milk production recording period.

### 2.4. Preoperative Management and Surgical Protocols

For all affected cows, the timeframe from LDA diagnosis to surgical intervention ranged between 24 and 36 h. Prior to each surgery, physical examinations were performed, and rectal temperature, respiratory rate, heart rate, and body condition score (BCS) were recorded on a field sheet. All surgical interventions were performed by a single skilled and experienced veterinarian (co-author of this paper). Preoperative preparation excluded fasting and sedation to prevent potential alterations in the pre-surgical blood parameters. All cows included in the study had returned to milk production. The mean rectal temperature, heart rate, respiratory rate, and BCS for cows in LPS and LPT groups were 38.4 ± 0.3 °C and 38.7 ± 0.4 °C, 77.8 ± 9.2 (beats/min) and 81.2 ± 8.7 (beats/min), 24.9 ± 4.6 (breaths/min) and 28.3 ± 5.2 (breaths/min), and 3.19 ± 0.2 and 3.16 ± 0.3 BCS, respectively.

The one-step laparoscopic abomasopexy was performed as follows: the surgical procedure was performed on a standing restrained cow, where, following aseptic preparation and local anesthesia, two portals were created to introduce the laparoscope and inspect the peritoneum, and access the abomasum. Under laparoscopic guidance, the greater curvature of the abomasum was cannulated to insert the safety toggle suture and deflate the organ. Finally, the abomasum was repositioned and fixed to the right ventral paramedian region using a Christiansen’s Spieker needle Dr. Fritz GmbH, Tuttlingen, Germany) guided by external palpation, with both ends of the suture secured externally over a gauze bandage for 15 days. The left paralumbar fossa omentopexy or the Utrecht approach was performed on restrained standing cow, where, after aseptic preparation and local infiltration anesthesia of the left flank, a 20–25 cm vertical incision was made in the left paralumbar fossa. The displaced abomasum was located, deflated using a sterile needle attached to a suction tube, abomasum manually repositioned beneath the rumen to its physiological position on the right side, and the greater omentum adjacent to the pylorus was anchored and fixed to the right ventral paramedian region with ends of sutures secured externally over a gauze bandage for 15 days.

Prior to incision, cows from both experimental groups were locally anesthetized using lidocaine hydrochloride (20 mg/ml) while postoperative protocol included administration of Procaine Benzylpenicillin 2,000,000 UI/Dihydrostreptomycin Sulphate 250 mg IM (Norbrook Laboratories Limited, Newry, UK) for three consecutive days, alongside a single IM administration of Tolfenamic Acid (40 mg/mL, 1 mL/20 kg of body weight) (Vetoquinol S.A., Lure, France). Postoperatively, all treated cows were maintained under identical group-housing conditions and offered the same fresh-cow TMR ad libitum. To monitor appetite recovery post-surgery, visual monitoring of feed refusals and clinical assessments of rumen fill scores were performed daily by the responsible veterinarian. All postoperative complications were limited to minor wound issues, which did not affect the healing process or the timing of suture removal.

### 2.5. Blood Samples and Analysis

Blood samples were taken from each cow by venipuncture of *v. caudalis mediana* immediately before treatment (D0), as well as on 1 (D1), 3 (D3), 10 (D10), and 30 (D30) days after treatment. Blood samples from the CON group were collected at the same time points. Samples for analysis of biochemical and hormonal variables were taken in gel-coated blood tubes (BD Vacutainer, Plymouth, UK) and allowed to clot spontaneously (approximately 20 min) on ice. After clotting, samples were centrifuged at 2000× *g* for 10 min to separate the serum, which was decanted into graduated polypropylene tubes (1.5 mL, Eppendorf AG, Hamburg, Germany) and then stored at −20 °C until analyses. All laboratory analyses were performed by personnel blinded to the experimental treatments and cow clinical status.

### 2.6. Metabolite and Hormone Analyses

Analyses of biochemical indicators included determination of glucose (mmol/L), beta-hydroxybutyrate (BHB; mmol/L), non-esterified fatty acids (NEFA; mmol/L), total bilirubin (μmol/L), aspartate aminotransferase (AST; U/L), gamma-glutamyl transferase (GGT; U/L), albumin (g/L), and uric acid (μmol/L). Analyses were performed at the Department of Ruminants and Swine Diseases (Belgrade, Serbia) automatically using a BioSystems S.A. biochemistry analyzer (Barcelona, Spain) and the respective commercial kits: NEFA (colorimetric method) and BHB (enzymatic method), both from Randox Laboratories Ltd. (Crumlin, UK); albumin (Bromcresol green method), total bilirubin (diazotized sulphanilic acid method), AST (IFCC method), GGT (IFCC method) and uric acid (uricase/peroxidase method) from BioSystems S.A. (Barcelona, Spain). Glucose concentration was measured in whole blood enzymatically (glucose dehydrogenase, GDH-NAD method) using commercial test strips (Abbott Diabetes Care Ltd., Oxon, UK). Serum insulin (uU/mL) levels were detected photometrically on an AIA 360 automated immunoassay analyzer (AIA-360, Tosoh Bioscience, Inc., South San Francisco, CA, USA) using commercially available competitive enzyme immunoassay kits from the same producer. The mean intra assay coefficient of variation (CV) for duplicate sample was 2.3%, while inter assay CV was <4.0%.

### 2.7. Estimation of Antioxidant Capacity in Blood Serum

The total antioxidant capacity (TAC) was evaluated by the method of 2.2-diphenyl-1-picrylhydrazyl (DPPH) radical scavenging ability. The estimation of TAC was done by measuring the reduction of DPPH radical spectrophotometrically according to Thaipong [32]; 50 μL of sample were mixed with 2.95 mL of DPPH solution and incubated in the dark for 1 h. The percentage of reduction was monitored by measuring the DPPH absorbance at 515 nm (the extent of the solution discoloration is proportional to the concentration of total antioxidants in the samples). A standard curve was calculated using a Trolox standard curve, according to the following equation: y = 2.78498 + 0.36837 ×, and the results are expressed as Trolox equivalents. The detection limit for the DPPH test is 1 micromol of Trolox, intra assay 1.9% and inter assay 3.5%.

### 2.8. Estimation of Paraoxonase 1 Activity in Blood Serum

The serum paraoxonase 1 (PON 1) activity was determined by measuring the initial rate of the synthetic paraoxon (diethyl-p-nitrophenyl phosphate) hydrolysis to p-nitrophenol. The absorbance was monitored at 412 nm in the assay mixture containing 2.0 mM paraoxon, 2.0 mM CaCl_2_, 5 M NaCl, and 40 μL of the serum sample. PON 1 activity was calculated using the molar extinction coefficient of phenol (18,500 M-1cm-1), and the results are presented as U/mL [33]. The detection limit for the PON1 test is 5 U/mL, intra assay 2.3%, and inter assay 8.1%.

### 2.9. Estimation of Myeloperoxidase Activity in Blood Serum

MPO activity in blood serum was determined spectrophotometrically at 550 nm, in the presence of o-dianisidine as a substrate in the reaction with H_2_O_2_, by monitoring the change in absorbance and the concentration of oxidized forms of o-dianisidine, according to the method described by Bradley [34]. MPO activity is expressed in U/mL. The detection limit of the MPO test is 5 U/mL, intra assay 2.5% and inter assay 5.2%.

### 2.10. Milk Production

Milk yield was recorded daily for all animals included in the study. Milk production data were retrieved daily from the central database of the on-farm herd management software analyzed and graphically presented for day 7 and at monthly intervals throughout the lactation period.

### 2.11. Data Analysis

Complete statistical analysis was performed in SPSS Statistics 29.0 software (IBM^®^, Armonk, NY, USA) and GraphPad Prism 8.0 (GraphPad Software Inc., San Diego, CA, USA). The normality of the data distribution was tested using the Shapiro–Wilk test. All data showed a normal distribution except for NEFA, MPO and PON1, which had to be logarithmically transformed (Log10). For NEFA, MPO, and PON1, which required logarithmic transformation due to non-normal distribution, the estimated marginal means and standard errors shown in the tables and figures were back-transformed to the original units. Data on the examined variables were analyzed using a linear mixed model with repeated measures. The model included fixed effects for surgical treatment (CON, LPS, and LPT), postoperative day (D1, D3, D10, and D30), the treatment × day interaction, and the baseline (D0) measurement as a covariate. Cow was included as a random effect, while day was considered as the repeated effect, and a first-order autoregressive repeated covariance structure:

*Y_ijk_* = *µ* + *T_i_* + *D_j_* + (*T* × *D*)*_ij_* + *β*_*Xik,*0_ + *u_ik_* + *ε_ijk_*

where *Y_ijk_* is the observed value of the dependent variable for cow *k* in treatment group *i* on day *j*; *μ* is the overall intercept; *T_i_* is the fixed effect of treatment group (CON, LPS, and LPT); *D_j_* is the fixed effect of sampling day (D1, D3, D10, and D30); (*T* × *D*)*_ij_* is the treatment group × day interaction; *Xik*,0 is the baseline (D0) value of the corresponding response variable for cow *k* in treatment group *i*; *β* is the regression coefficient associated with the baseline covariate; *u_ik_* is the random intercept for cow *k* in treatment group *i*; and *ε_ijk_* is the residual error. When the linear mixed model indicated significant effects, the Tukey procedure was used for pairwise comparisons. All data in tables and figures are expressed as estimated marginal means ± standard error (EMM ± SE).

Significance was declared at *p* < 0.05, *p* < 0.01, and *p* < 0.001.

## 3. Results

The effects of surgical treatment for a left-displaced abomasum (LDA) on energy metabolism, liver function, antioxidant status, and inflammatory response indices in dairy cows are reported in Table 2. Pairwise differences in estimated marginal means between groups at each sampling day, with 95% confidence intervals and Tukey-adjusted *p*-values are provided in Supplementary Table S1.

### 3.1. Blood Biochemical Indices

#### 3.1.1. Energy Metabolism

**Figure 1:**
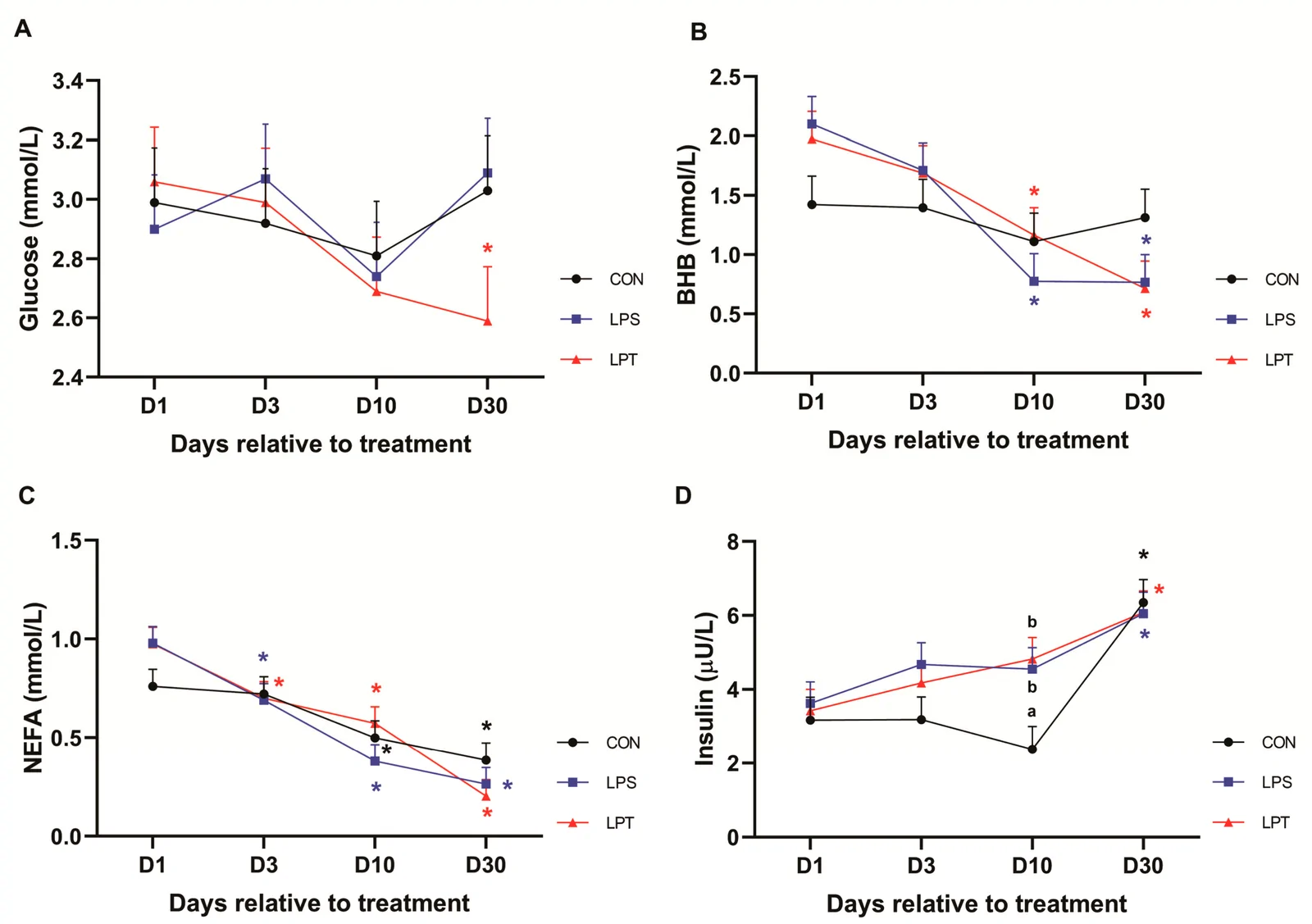
Temporal changes (EMM ± SE) in indices associated with energy metabolism ((**A**)—Glucose; (**B**)—BHB; (**C**)—NEFA; (**D**)—Insulin) in CON, LPS, and LPT cows. CON—control group; LPS—laparoscopy group; LPT—laparotomy group. ^a,b^—different lowercase letters indicate a significant difference (*p* < 0.05) between groups at the same time point. *—Asterisk indicates a significant difference (*p* < 0.05) within group in days after treatment (D3, D10, D30) compared to the D1.

Figure 1 shows that surgical treatment for LDA did not significantly affect any energy metabolism indicator (*p* > 0.050), whereas postoperative day significantly influenced BHB (*p* < 0.001), NEFA (*p* < 0.001), and insulin (*p* < 0.001) concentrations. Accordingly, BHB and NEFA concentrations progressively decreased, while insulin concentrations increased from the first (D1) to the thirtieth (D30) postoperative day. The treatment × day interaction significantly affected BHB concentrations (*p* = 0.037) and NEFA concentrations (*p* = 0.045).

#### 3.1.2. Liver Function

**Figure 2:**
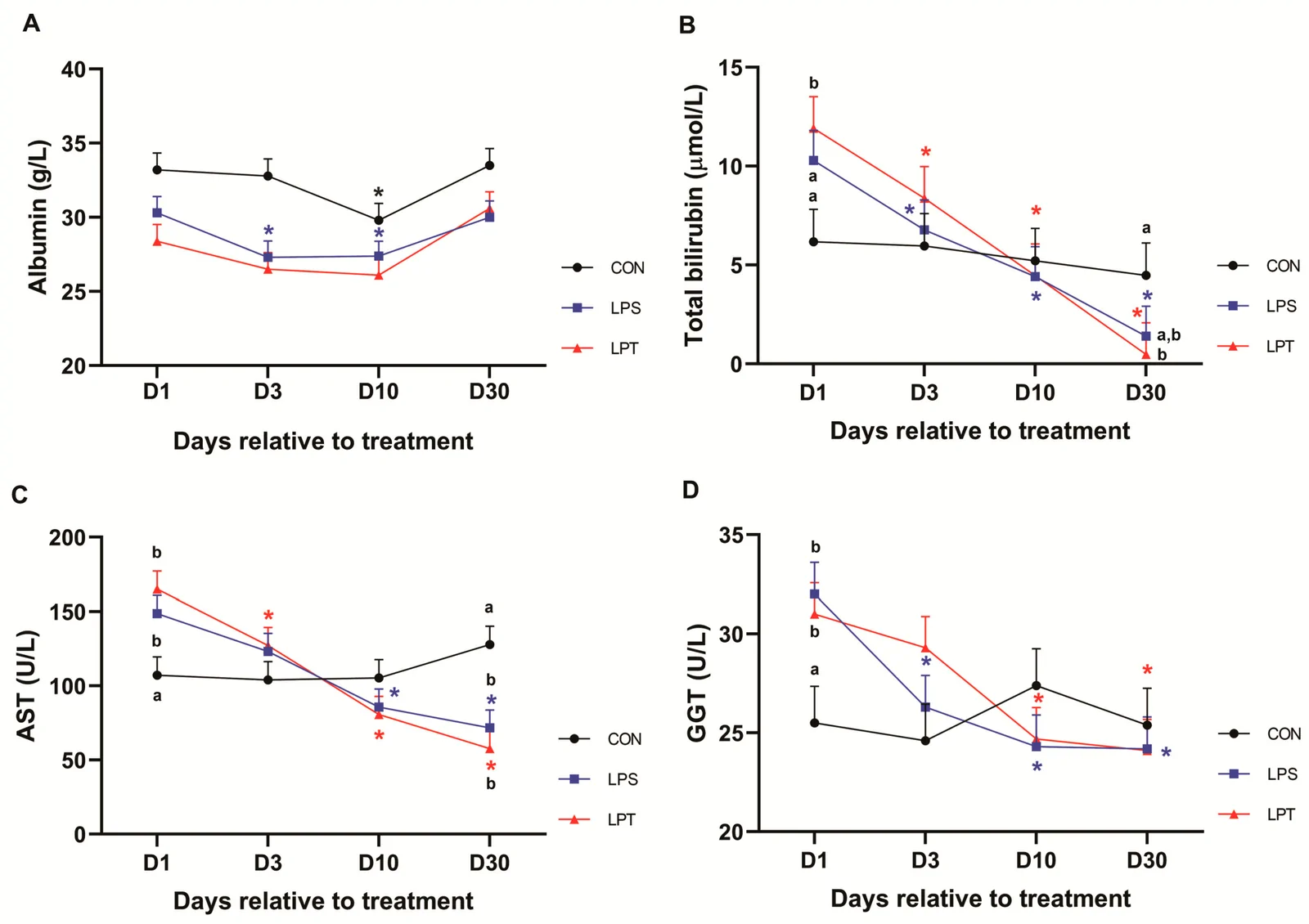
Temporal changes (EMM ± SE) in indices associated with liver function ((**A**)—Albumin; (**B**)—Total bilirubin; (**C**)—AST; (**D**)—GGT) in CON, LPS, and LPT cows. CON—control group; LPS—laparoscopy group; LPT—laparotomy group. ^a,b^—different lowercase letters indicate a significant difference (*p* < 0.05) between groups at the same time point. *—Asterisk indicates a significant difference (*p* < 0.05) within group in days after treatment (D3, D10, D30) compared to D1.

Regarding liver function indices, surgical treatment for LDA significantly affected albumin concentrations (*p* = 0.030) and AST activity (*p* = 0.022), while total bilirubin concentrations (*p* = 0.067) and GGT activity (*p* = 0.074) tended to be affected. Therefore, both LPS and LPT cows had significantly lower albumin concentrations than CON cows (*p* = 0.005, *p* = 0.001, respectively). All reported liver function indices were significantly affected by postoperative day (*p* < 0.001). Namely, total bilirubin concentrations and AST and GGT activities successively declined from D1 to D30. A similar pattern was observed for albumin concentration from D1 to D10, but with a sharp rise on D30. Interestingly, significant treatment × day interactions were found for total bilirubin concentrations (*p* = 0.019), AST (*p* < 0.001), and GGT (*p* = 0.011) activities. Tukey-adjusted comparisons showed that total bilirubin concentrations were significantly higher on D1 (*p* = 0.025) and tended to be lower on D30 (*p* = 0.030) in LPT than in CON cows. In addition, both LPS and LPT cows had higher activities of AST (*p* = 0.037; *p* = 0.007; respectively) and GGT (*p* = 0.028; *p* = 0.054; respectively) than CON cows on D1, whereas on D30, only the activity of AST was significantly lower in both LPS and LPT cows compared to CON cows (*p* = 0.007; *p* = 0.002; respectively) (Figure 2).

### 3.2. Antioxidant Status and Inflammatory Response

**Figure 3:**
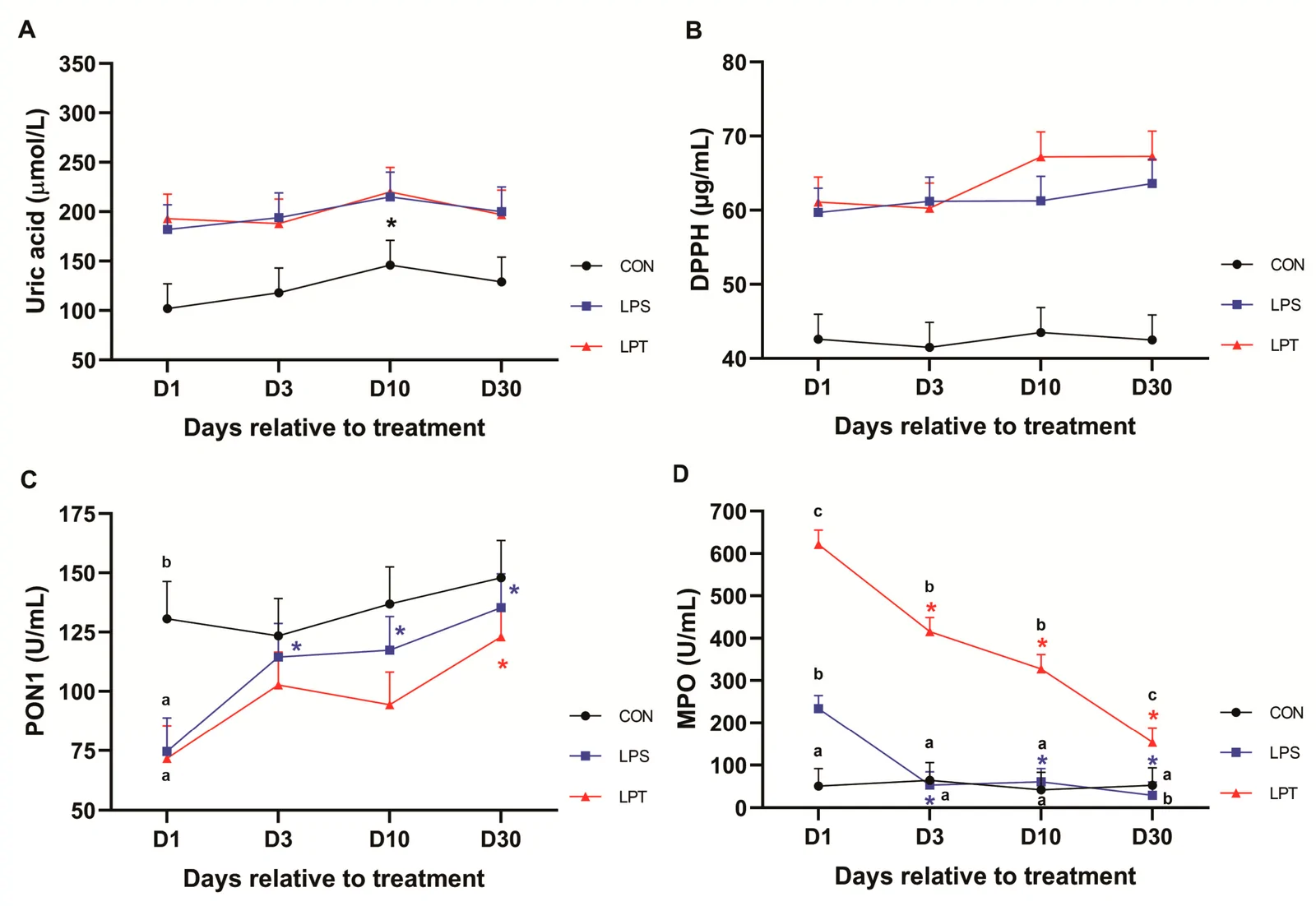
Temporal changes (EMM ± SE) in indices associated with antioxidant status and inflammatory response ((**A**)—Uric acid; (**B**)—DPPH; (**C**)—PON1; (**D**)—MPO) in CON, LPS, and LPT cows. CON—control group; LPS—laparoscopy group; LPT—laparotomy group. ^a,b,c^—different lowercase letters indicate a significant difference (*p* < 0.05) between groups at the same time point. *—Asterisk indicates a significant difference (*p* < 0.05) within group in days after treatment (D3, D10, D30) compared to D1.

For indices associated with antioxidant status and inflammatory response, surgical treatment for LDA significantly affected uric acid (*p* = 0.040), DPPH (*p* = 0.002), PON1 (*p* = 0.016), and MPO (*p* < 0.001) levels (Figure 3). Thus, LPS and LPT cows had higher concentrations of uric acid (*p* = 0.068; *p* = 0.056; respectively) and DPPH (*p* < 0.001; *p* < 0.001; respectively) than CON cows. Moreover, MPO activity was significantly lower in both LPS (*p* < 0.001) and CON (*p* < 0.001) cows than in LPT cows. Interestingly, MPO levels were significantly affected by the treatment × day interaction (*p* < 0.001), revealing significantly higher values in both LPS (*p* < 0.001) and LPT (*p* < 0.001) cows compared to CON on D1, but also significantly lower values in LPS than in LPT cows (*p* < 0.001) on the same postoperative day. In addition, Tukey-adjusted comparisons showed that both LPS and CON cows exhibited significantly lower MPO levels than LPT cows on D3 (*p* < 0.001; *p* < 0.001; respectively), D10 (*p* < 0.001; *p* < 0.001; respectively), and D30 (*p* < 0.001; *p* = 0.008; respectively) (Figure 3). Moreover, MPO activities were significantly lower in LPS than in CON cows on D30 (*p* < 0.001). Besides the effect of the treatment × day interaction on MPO activities, tendencies were observed for PON1 activities (*p* = 0.099), revealing significantly lower values in both LPS and LPT cows on D1 than in CON cows (*p* = 0.011; *p* = 0.008; respectively). Finally, postoperative day significantly affected uric acid (*p* = 0.046), PON1 (*p* = 0.003), and MPO (*p* < 0.001). Uric acid concentrations increased until D10 and then declined by D30, whereas PON1 activity increased throughout the postoperative period. In contrast, MPO activity progressively decreased from D1 to D30, indicating that uric acid and PON1 increased while MPO rapidly decreased.

### 3.3. Dynamics of Milk Yield

**Figure 4:**
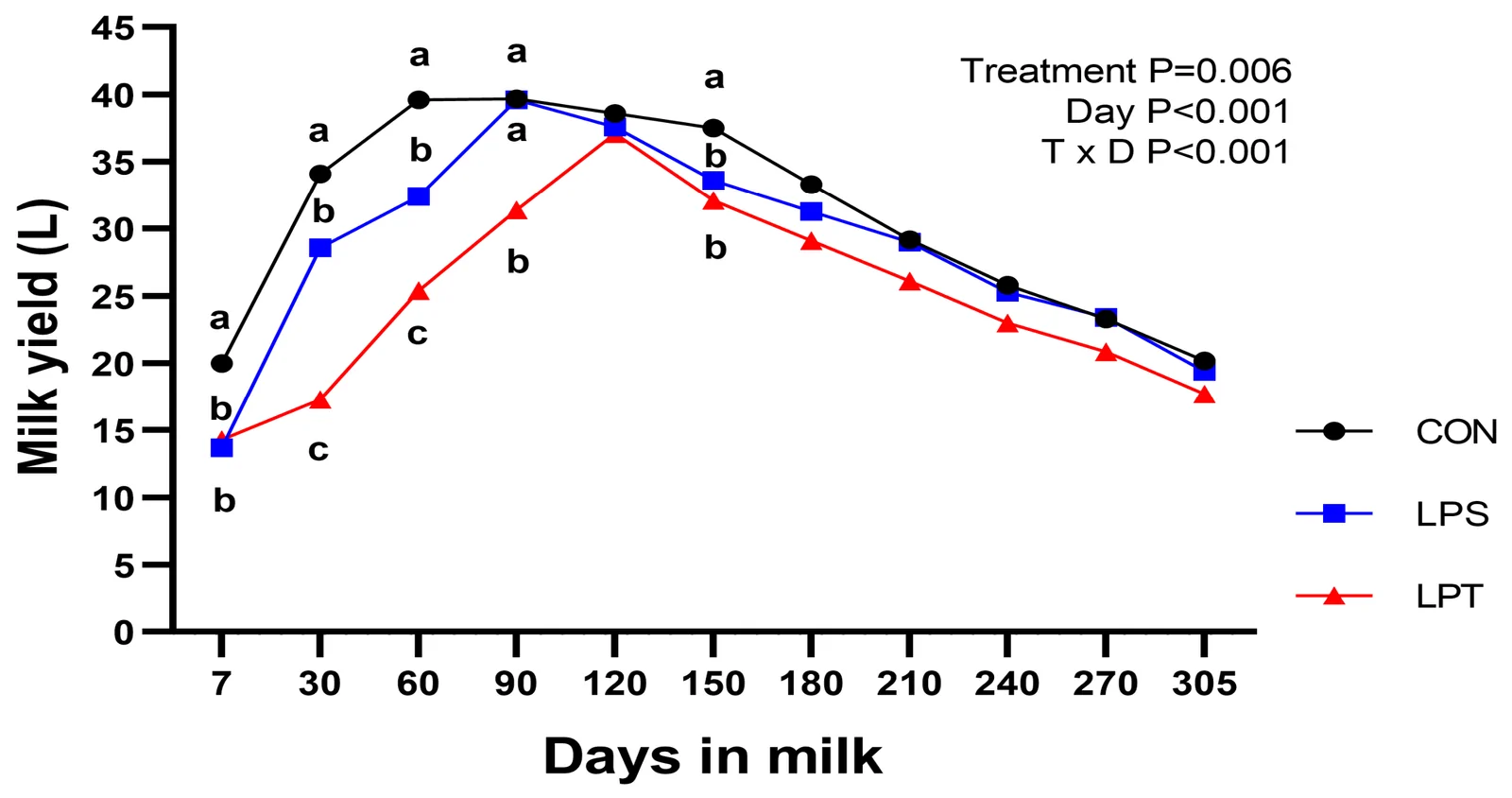
Monthly dynamics of milk production in CON, LPS, and LPT cows. CON—control group; LPS—laparoscopy group; LPT—laparotomy group. ^a,b,c^—different lowercase letters indicate a significant difference (*p* < 0.05) between groups at the same time point.

The monthly dynamics of milk yield in CON, LPS, and LPT cows are shown in Figure 4. There was a significant influence of treatment (*p* = 0.006), day (*p* < 0.001) and treatment × day interaction (*p* < 0.001). Compared to CON cows, milk yield was significantly lower in both LPS and LPT on days 7 (*p* = 0.037; *p* = 0.048; respectively), 30 (*p* = 0.007; *p* < 0.001; respectively) and 60 (*p* = 0.009; *p* < 0.001; respectively) of lactation. Moreover, LPT cows had significantly lower milk yield than LPS cows on days 30 (*p* < 0.001) and 60 (*p* = 0.016). Interestingly, LPS cows matched milk yield to CON cows on day 90, while LPT cows had significantly lower milk yield than CON (*p* < 0.001) and LPS (*p* = 0.005) cows on the same lactation day. Although the groups of cows studied showed comparable milk yield on day 120, a significant decrease in milk yield was observed in both LPS (*p* = 0.042) and LPT (*p* = 0.012) compared to CON cows on day 150. Finally, from day 180 to day 305, there were no significant differences (*p* > 0.05) between CON, LPS, and LPT cows. When considering the entire 305-day lactation, LPS cows had 6.84% lower total milk yield (8898 ± 588 L) than CON cows (9551 ± 396 L), while LPT cows exhibited 11.79% (8425 ± 280 L) lower total milk yield compared to CON cows with a statistical significance (*p* = 0.019).

## 4. Discussion

The results of this study demonstrate the beneficial effects of laparoscopic correction of LDA compared with left paralumbar fossa omentopexy (Utrecht approach) in reducing postoperative stress response and preserving milk production in multiparous dairy cows. This is the first study to demonstrate the advantages of laparoscopy in assessing postoperative MPO activity in the blood of multiparous dairy cows. The occurrence rate of LDA in multiparous cows (81.2%) on the selected farm concurs with findings from previous studies, indicating that the risk of developing a DA increases with age [4]. As described previously, the majority of LDA cases diagnosed in this study occurred within four weeks and peaked at approximately two weeks after calving [8].

The metabolic profiles in serum samples from both LDA cows and the healthy control group indicate metabolic overload in LDA cows and differential postoperative dynamics of indices targeting energy metabolism and hepatic function between the two surgical groups. Research studies on blood glucose levels in LDA cows have yielded inconsistent findings. Namely, some studies have reported increased blood glucose levels in cows with LDA [2,35], whereas others have not [36,37]. In the present study, blood glucose concentrations were not significantly affected by the type of surgical treatment (*p* = 0.817), nor did they exhibit a significant treatment × day interaction (*p* = 0.472). Abdominal surgeries trigger a hypermetabolic response in cattle often leading to transient alterations in systemic glycemia [38]. Although our previous study on primiparous cows [8] demonstrated significantly lower immediate postoperative cortisol levels in laparoscopically treated compared to cows treated by left paralumbar fossa omentopexy, glucose and insulin levels in the multiparous cows evaluated herein remained uniform across both surgical groups. Similar to changes in blood glucose concentrations, insulin levels in this study in both LPT and LPS cows were lower on the first day after surgery compared to the CON group, arguing against a compromised insulin resistant state in these cows. The postoperative hypoinsulinemia found in cows with LDA in this trial was most likely a result of decreased feed intake, as supported by the findings of Stengärde et al. [36] and Song et al. [37]. Moreover, insulin is a critical driver for body reserve mobilization in early-lactation cows, and thus lower levels of insulin help explain the higher adipose lipolytic rate, i.e., the higher NEFA levels, and the higher concentrations of BHB in the LPS and LPT cows on D1 relative to treatment. Likewise, on D1 and D3, evidence of heightened NEB characterized by intense lipomobilization and ketogenesis was obvious both for LPT and LPS cows with NEFA and BHB concentrations elevated above the upper limits (>0.6 mmol/L and >1.2 mmol/L; respectively). Although both LPS and LPT cows significantly decreased their NEFA and BHB levels at D10, the values of these indices returned within physiological ranges on D10 only in LPS cows, suggesting a favorable metabolic response in these cows.

The lipomobilization effect and surgical trauma are known to trigger metabolic inflammation, which impact hepatic metabolism, impairing liver function and/or diverting synthesis in the liver during inflammation. In the present study, this condition is consistent with the pathological levels of AST and GGT alongside the levels of bilirubinemia found in both LPT and LPS cows during the early postoperative period. Although the main effect of treatment did not reach statistical significance for total bilirubin (*p* = 0.067) and GGT activity (*p* = 0.074), a highly significant treatment × day interaction was established for both parameters (*p* = 0.019 and *p* = 0.011; respectively). This interaction showed that the postoperative bilirubin recovery patterns and GGT stabilization differed significantly between the surgical groups. Total bilirubin concentrations were significantly higher on D1 in LPT cows compared to both the LPS and CON groups (*p* = 0.031). This early post-partum spike could point to a possible more severe impairment of the liver’s excretory capacity immediately following conventional laparotomy. At the same time, on D3, bilirubin concentrations returned within the normal range (0–8.5 µmol/L; Kaneko [39]) in LPS cows. The distinct temporal resolution of bilirubin levels within physiological ranges in LPS cows aligns with the findings of Seeger et al. [11], who also reported a faster normalization of excretory capacity after laparoscopic LDA correction. However, this parameter is considered to be pathological when greater than 8.55 µmol/L and as a consequence of temporary reduction in the liver’s excretory capacity and increased hepatic lipid accumulation. In addition, the postoperative course of serum GGT levels showed a significant decrease in GGT activity in the LPS group at D3 compared to the D1, while this was not the case in the LPT group. A more pronounced fall in blood GGT levels in LPS compared to LPT cows could possibly reflect a response to the severity of surgical stress and/or oxidative stress [40]. Likewise, the positive effects exerted by laparoscopy on hepatic lipid metabolism and inflammatory conditions could account for the faster recovery of GGT levels within the reference values found at D3 in LPS cows compared to LPT cows. Moreover, a decrease in the liver synthesis of negative acute phase proteins also occurs during the systemic inflammatory response; thus, the hypoalbuminemia found before and after surgery in LDA cows can be caused, at least in part, by the systemic inflammation that occurred in these cows. Non-significant treatment × day interaction for serum albumin (*p* = 0.579) showed a uniform postoperative trend across both surgical techniques. Therefore, the general suppression of albumin synthesis most probably reflects a response to the baseline metabolic stress of LDA rather than a procedure-specific deviation in capillary permeability or fluid shifts at D1. Conversely, the significant main effects and interaction for AST activity (*p* = 0.022 and *p* < 0.001) demonstrated that the extent of hepatic metabolic stress and the subsequent rate of its postoperative recovery were specifically modulated by the applied surgical method.

The assessment of serum antioxidant system status during pre- and postoperative periods is valuable for evaluating recovery from abdominal surgeries in cattle, as shown previously for LDA [28,30]. However, changes in oxidative and antioxidant biomarkers during the postoperative period of LDA cows in relation to different surgical approaches and/or types of injury are relatively unexplored. Furthermore, inconsistent findings have been reported regarding the total antioxidant levels in cows with LDA. Contrary to studies by Aly et al. [41] and Mirzaei et al. [22], no significant differences in TAC were found between cows with LDA and healthy controls during the postoperative period in our study. Unexpectedly, we did not measure a decrease in TAC value after surgery, since both the LPT and LPS cows responded by raising their serum antioxidant capacity. Our findings align with those of Maden et al. [26] and Durgut et al. [42], who also did not observe reduced antioxidant capacity in cows with LDA, implying that multiple factors and/or analytical methods may influence the antioxidant status of LDA cows. In the present study, we used the DPPH test as a marker for the total antioxidant capacity in serum. Since the DPPH assay reflects the overall radical-scavenging capacity of serum, this increase may indicate an enhanced ability of circulating antioxidants to neutralize free radicals during the postoperative period. Such a response may represent an adaptive response of the endogenous antioxidant defense system, particularly through changes in circulating non-enzymatic antioxidants, including albumin, uric acid, bilirubin, and other low-molecular-weight antioxidants that substantially contribute to serum antioxidant capacity [43,44]. A postoperative increase in serum antioxidant capacity, as observed in the present study, has not been previously reported in dairy cows undergoing LDA surgery, and the mechanisms underlying this response remain unclear. Interestingly, overall postoperative DPPH remained high in both surgical groups relative to the CON group; however, none of the individually measured non-enzymatic parameters exhibited significant differences when LPS and LPT groups were directly compared. While postoperative albumin and uric acid profiles along with the short-term increase in total bilirubin coincided with the overall elevation in DPPH, their similar recovery profiles could indicate a shared compensatory response to post-surgical oxidative stress across both trial groups, rather than a variation in LDA surgery technique. In addition, these findings could imply an additive or synergistic effect of circulating molecules responsible for antioxidant defense, consistent with the integrated nature of the bovine non-enzymatic antioxidant network [44,45]. Furthermore, the rise in DPPH without significant differences in individually measured indices could potentially have been complemented by other unmeasured endogenous radical scavengers, such as glutathione or vitamins E, A, and C. These molecules are known to undergo dynamic mobilization during the post-surgical oxidative stress in high-producing dairy cows [46].

PON1, a high-density lipoprotein (HDL)-associated enzyme synthesized in the liver, is used as a reliable indicator of oxidative stress and liver function disturbance in periparturient dairy cows [47,48]. It has been shown that tissue damage caused by the tension in displaced abomasum can elicit an inflammatory response [26] followed by a reduction in PON1 activity [49]. The results of the present study show significant attenuation of PON1 activity at D1 in LPT and LPS groups compared to the control group, as previously reported by Aslan et al. [28]. Furthermore, a significant increase in PON1 activity relative to D1 was observed starting from D3 in the LPS group, whereas the LPT group required a 30-day period to exhibit a similar recovery profile. The observed differences in the PON1 recovery pattern between the LPS and LPT groups coincide with the postoperative dynamics of the pro-oxidant MPO, which exhibited significantly lower levels in laparoscopically treated animals compared to the LPT group. Presumably, less invasive surgical approaches such as laparoscopy limit inflammation and MPO secretion, thereby sparing systemic PON1 activity. This inverse relationship between these two enzymes aligns with previous reports [50]. In addition, PON1 levels at D1 were accompanied by elevated NEFA and BHB levels in LDA cows, which are known to provoke oxidative stress and/or reduce PON1 serum activity [48]. In other words, a significant reduction in NEFA and BHB, during the postoperative period in this trial could attenuate the oxidative burden on hepatocytes, potentially enhancing PON1 activity across both surgical groups [27,28].

The most distinct differences between the two approaches for LDA surgery were recorded for serum MPO activity, which exhibited significant main effects for treatment (*p* < 0.001), day (*p* < 0.001), and a treatment × day interaction (*p* < 0.001). MPO, an enzyme released by activated neutrophils during an inflammatory response, catalyzes the formation of ROS, and its elevated levels are used as biomarkers of neutrophil infiltration and subsequent oxidative damage in situations of surgical tissue trauma, inflammation, and sepsis [51,52]. A study investigating the effects of prepartum body condition score (BCS) on oxidative stress during the transition period reported an increase in MPO activity in high BCS cows [53], and previous research has also shown that the increase in MPO is associated with greater inflammation and oxidative stress in Holstein cows [54,55]. In a study with horses, Grulke et al. [56] observed that plasma MPO levels increased in response to pathological displacement of intestines and accompanying inflammation. Based on a lower pro-inflammatory response, Gomez-Pinilla et al. [57] concluded that mice operated under laparoscopic conditions had a faster recovery and significantly lower intestinal inflammation compared to those treated by laparotomy. The available literature lacks information on monitoring changes in blood MPO levels in response to different surgical LDA treatment options in affected dairy cows. Therefore, this is the first study to describe MPO activity during the postoperative period following laparoscopic LDA surgery and to assess the significance of MPO as a biomarker of inflammation and stress compared with laparotomy techniques. In the present study, we found increased early post-partum activity of serum MPO in both the LPS and LPT groups compared with the CON. This is in accordance with differences in MPO activity between cows with a DA and healthy cows reported by Maden et al. [26], who explained the enhanced level of MPO activity through changes in the severity of inflammation and tissue necrosis while the abomasum is displaced [26]. In addition, elevated levels of MPO in LPT cows persisted until D30 compared to both LPS and CON, while it resolved in the LPS group to a level close to that measured in CON cows by D3. These findings could indicate lower inflammatory cell activity and consequently less tissue damage after laparoscopic LDA surgery compared to the classical Utrecht approach. In support of this, a study by Costa et al. [58] compared two surgery approaches to canine ovariectomy and found increased MPO and malondialdehyde levels in the open surgery group compared to the laparoscopic group. Overall, according to the post-surgery PON-1 and MPO dynamics in the present study, laparoscopic LDA surgery showed clear advantage in terms of faster short-term recovery due to a lower inflammation response and/or reduced oxidative stress compared to classic left paralumbar omentopexy.

Some estimates are that the average milk loss per lactation from a LDA, due to the direct reduction in milk production and the residual effects during the convalescence period, falls between 7.5% and 11% [4]. Given the fact that LPT cows in the present trial suffered the prolonged inability of the liver to perform proper metabolic regulation, overall lactation in these cows, as expected, was lower compared to their control herd-mates. At the same time, the 305-day milk yield between group LPS and group CON approached and did not reach statistical significance. Conversely, Fiore et al. [59] reported that cows treated with 2-step laparoscopic abomasopexy produced less milk over 305 days compared to the healthy control group. This difference might be explained by the varying laparoscopic methods used in each study. Namely, placing cows in lateral recumbency, which is a standard procedure during the 2-step but not for the 1-step method, may have the potential to facilitate injury and/or result in a stress-induced increase in blood cortisol [60], which in turn could delay the return to optimal milk production. In the present study, we found marked preoperative reduction in milk production in both LPS and LPT groups compared to CON. Moreover, the lower milk production extended to 120 DIM in LPT cows, while LPS cows reached the production level of their matched controls in the third month of lactation. Furthermore, LPS cows had higher production levels at 30–90 DIM when compared to LPT cows. These results are in agreement with our previous study [8], where better lactational performance recovery was observed in response to laparoscopic surgery in primiparous cows. In Giannuzzi et al.’s [46] study, increasing levels of oxidative stress metabolites, such as MPO, as well as the reduction of negative acute phase protein PON1, were shown to be associated with reduction in milk yield. Taken together, the lactational responses of LDA cows to the two surgical methods indicate that laparoscopic surgery may support a more favorable trend in short-term milk production recovery compared with laparotomic treatment. These findings can, at least in part, be related to much less surgical trauma and a reduced burden on the liver function in LPS cows.

There are several limitations to the present study. First, the experimental phase, encompassing diagnosis, LDA surgery, and intensive sampling, was restricted to a 4-month period (March–June) to minimize potential seasonal compounding effects on LDA occurrence. Consequently, the relatively small sample size per group represented the maximum feasible number that fulfilled all inclusion and health criteria outlined in Section 2. Second, due to the constraints of conducting this research within a high-producing commercial facility, more specialized investigation of the particular mechanistic pathways explaining the role of MPO in post-surgical inflammation and its association with milk production was hard to conduct in the scope of this study. Likewise, the lack of a longitudinal regression analysis in our study limits the statistical confirmation of a predictive relationship between these specific biomarkers and milk yield. Thus, further statistical analyses are required to fully quantify their longitudinal predictive capacity in terms of milk production.

## 5. Conclusions

In conclusion, our findings highlight MPO as a promising candidate for evaluating post-surgical trauma and systemic inflammation in LDA cows. Although further large-scale validation is required, the descriptive alignment between postoperative increase in inflammatory and oxidative stress markers, such as MPO, and changes in milk yield follow a similar time-course pattern. Therefore, laparoscopy in one step is not only important to ameliorate the postoperative inflammatory response, reflected in changes in circulatory MPO and PON1, but also to support a more rapid short-term recovery of milk production, specifically during early lactation (30–90 DIM). In addition, postoperative dynamics of energy metabolism and liver-related biochemical variables were more favorable with laparoscopy, confirming the expediency of LDA laparoscopic treatment under field conditions. Further studies are needed to fully elucidate the specific mechanisms by which hepatocytes respond to laparoscopic correction under these conditions.

## Figures and Tables

**Table 1:** Ingredients and chemical composition of the diet for cows in early lactation.

Diet	Early Lactation
**Ingredient (g/kg of DM)**	
Corn silage	410
Alfalfa silage	120.81
Alfalfa hay	5.2
Molasses	33.95
Corn grain	139.4
Barley	59.74
Soybean meal (44% CP)	97.76
Sunflower meal (35% CP)	92.6
Sodium chloride	3.15
Sodium bicarbonate	5.4
Calcium carbonate	9.01
Vitamin Mineral Mix	4
Protected fat (99% palmitic acid)	18.98
Chemical composition	
NEL (Mcal/kg of DM)	1.71
CP (g/kg of DM)	166.7
RDP (g/kg of CP)	106.0
RUP (g/kg of CP)	60.7
MP (g/kg of DM)	106.27
aNDF (g/kg of DM)	304.79
ADF (g/kg of DM)	197.56
NFC (g/kg of DM)	408.31
Ether Extract (g/kg of DM)	45
Ca (g/kg of DM)	8.99
P (g/kg of DM)	4.19

DM—Dry matter. CP—Crude protein. NEL—Net energy for lactation. RDP—Rumen degradable protein. RUP—Rumen undegradable protein. MP—Metabolizable protein. aNDF—Amylase-treated neutral detergent fiber. ADF—Acid detergent fiber. NFC—Non-fiber carbohydrate.

**Table 2:** The effects of surgical treatment for a left-displaced abomasum (LDA) on energy metabolism, liver function, antioxidant status, and inflammatory response indices in dairy cows.

Parameter	Treatment (T)			SE	Days (D)				SE	*p*-Value		
	CON	LPS	LPT		D1	D3	D10	D30		T	D	T × D
Glucose (mmol/L)	2.94	2.95	2.83	0.126	2.98	3.00	2.75	2.90	0.106	0.817	0.159	0.472
BHB (mmol/L)	1.31	1.34	1.39	0.175	1.83 ^a^	1.60 ^a^	1.02 ^b^	0.93 ^b^	0.132	0.148	<0.001	0.037
NEFA (mmol/L)	0.592	0.579	0.613	0.055	0.905 ^a^	0.704 ^a^	0.484 ^b^	0.284 ^c^	0.048	0.655	<0.001	0.045
Insulin (µIU/mL)	3.77	4.73	4.63	0.336	3.40 ^a^	4.01 ^a^	3.92 ^a^	6.16 ^b^	0.330	0.873	<0.001	0.231
Albumin (g/L)	32.3 ^a^	28.7 ^b^	27.9 ^b^	0.668	30.6 ^a^	28.9 ^b^	27.7 ^b^	31.4 ^a^	0.636	0.030	<0.001	0.579
T-bilirubin (µmol/L)	5.62	5.70	5.94	0.868	9.46 ^a^	7.04 ^b^	4.70 ^c^	1.80 ^d^	0.832	0.067	<0.001	0.019
AST (IU/L)	111.0	107.0	108.0	5.820	140.3 ^a^	118.1 ^b^	90.6 ^c^	85.8 ^c^	6.930	0.022	<0.001	<0.001
GGT (IU/L)	25.7	26.7	27.3	1.125	29.5 ^a^	26.7 ^b^	25.5 ^b^	24.6 ^b^	0.868	0.074	<0.001	0.011
Uric acid (µmol/L)	124 ^a^	198 ^b^	200 ^b^	21.40	159 ^a^	167 ^a^	193 ^b^	175 ^ab^	14.40	0.040	0.046	0.987
DPPH (%)	42.5 ^a^	61.4 ^b^	64.0 ^b^	2.610	54.5	54.3	57.3	57.8	1.890	0.002	0.230	0.717
PON1 (U/L)	134.8	110.5	98.0	10.78	92.3 ^a^	113.6 ^ab^	116.2 ^b^	135.5 ^b^	7.730	0.016	0.003	0.099
MPO (U/L)	52.6 ^a^	94.3 ^a^	379.6 ^b^	25.20	302.1 ^a^	177.9 ^b^	143.5 ^b^	78.6 ^c^	16.50	<0.001	<0.001	<0.001

Main effects of treatment (T), day (D), and treatment × day interaction (T × D). ^a,b,c,d^—estimated marginal means with different superscript letters within a row differ significantly (*p* < 0.05) according to Tukey pairwise comparisons.

## Data Availability

The original contributions presented in this study are included in the article. Further inquiries can be directed to the corresponding author.

## Supplementary Materials

The following supporting information can be downloaded at https://www.mdpi.com/article/10.3390/ani16213366/s1. Table S1: Pairwise differences in estimated marginal means between groups at each sampling day, with 95% confidence intervals and Tukey-adjusted *p*-values.
