# Supplementary material for: Effects of One-Step Laparoscopic Abomasopexy and Laparotomic Omentopexy Treatments of Left Displaced Abomasum on Metabolic and Inflammatory Stress Responses, Liver Health and Milk Yield in Multiparous Cows
Source: Animals (Basel). 2026 Oct 25;16(21):3366. doi: 10.3390/ani16213366 (PMC13761560; doi:10.3390/ani16213366)
Supplement: Supplementary file 1 [file animals-16-03366-s001.zip › animals-4566174-supplementary.pdf]

Table S1. Pairwise differences in estimated marginal means between groups at each sampling day, with 95% confidence intervals and Tukey-adjusted P-values.

Parameter	Day	Pairwise Comparisons	EMM difference	95% CI for the difference		Tukey-adjusted *P* values
				Lower	Upper	
Glucose (mmol/L)	D1	LPS – CON	-0.0848	-0.632	0.463	0.748
		LPT – CON	0.0799	-0.467	0.626	0.762
		LPT – LPS	0.1646	-0.377	0.7064	0.530
	D3	LPS – CON	0.1524	-0.395	0.700	0.565
		LPT – CON	-0.1101	-0.657	0.436	0.676
		LPT – LPS	-0.0468	-0.589	0.4949	0.858
	D10	LPS – CON	-0.0633	-0.611	0.484	0.810
		LPT – CON	0.0727	-0.474	0.619	0.782
		LPT – LPS	-0.0797	-0.621	0.4621	0.760
	D30	LPS – CON	0.0638	-0.484	0.611	0.809
		LPT – CON	-0.4344	-0.981	0.112	0.112
		LPT – LPS	-0.4982	-1.040	0.0435	0.069
BHB (mmol/L)	D1	LPS – CON	0.6774	-0.0462	1.401	0.065
		LPT – CON	0.5537	-0.1690	1.276	0.124
		LPT – LPS	-0.1237	-0.804	0.5572	0.706
	D3	LPS – CON	0.3259	-0.3977	1.050	0.355
		LPT – CON	0.2923	-0.4304	1.015	0.405
		LPT – LPS	-0.0337	-0.714	0.6472	0.918
	D10	LPS – CON	-0.3341	-1.0577	0.390	0.344
		LPT – CON	0.0551	-0.6676	0.778	0.874
		LPT – LPS	0.3892	-0.292	1.0700	0.244
	D30	LPS – CON	-0.5455	-1.2691	0.178	0.130
		LPT – CON	-0.5977	-1.3204	0.125	0.099
		LPT – LPS	-0.0522	-0.733	0.6286	0.873
NEFA (mmol/L)	D1	LPS – CON	0.0795	-0.136	0.2951	0.447
		LPT – CON	0.0904	-0.130	0.3113	0.400
		LPT – LPS	0.01087	-0.19241	0.2142	0.911
	D3	LPS – CON	-0.0398	-0.255	0.1757	0.701
		LPT – CON	-0.0332	-0.254	0.1878	0.755
		LPT – LPS	0.00669	-0.19659	0.2100	0.945
	D10	LPS – CON	-0.1212	-0.337	0.0944	0.252
		LPT – CON	0.0738	-0.147	0.2947	0.490
		LPT – LPS	0.19498	-0.00830	0.3983	0.059
	D30	LPS – CON	-0.0593	-0.275	0.1562	0.569
		LPT – CON	-0.2003	-0.421	0.0206	0.073
		LPT – LPS	-0.14104	-0.34433	0.0622	0.161
Insulin (µIU/mL)	D1	LPS – CON	0.459	-1.395	2.31	0.608
		LPT – CON	0.257	-1.607	2.12	0.775
		LPT – LPS	-0.2020	-1.91	1.502	0.806
	D3	LPS – CON	1.501	-0.352	3.35	0.106
		LPT – CON	0.999	-0.864	2.86	0.274
		LPT – LPS	-0.5020	-2.21	1.202	0.543
	D10	LPS – CON	2.173	0.320	4.03	0.024
		LPT – CON	2.442	0.578	4.31	0.013
		LPT – LPS	0.2694	-1.44	1.974	0.743
	D30	LPS – CON	-0.306	-2.159	1.55	0.732
		LPT – CON	-0.272	-2.136	1.59	0.762
		LPT – LPS	0.0337	-1.67	1.738	0.967
Albumin	D1	LPS – CON	-2.95	-6.33	0.433	0.083

(g/L)		LPT – CON	-4.79	-8.24	-1.337	0.009
		LPT – LPS	-1.839	-5.133	1.46	0.255
	D3	LPS – CON	-5.47	-8.85	-2.090	0.003
		LPT – CON	-6.28	-9.73	-2.826	0.001
		LPT – LPS	-0.804	-4.098	2.49	0.613
	D10	LPS – CON	-2.38	-5.77	0.997	0.155
		LPT – CON	-3.70	-7.15	-0.247	0.037
		LPT – LPS	-1.313	-4.607	1.98	0.412
	D30	LPS – CON	-3.55	-6.93	-0.166	0.041
		LPT – CON	-2.96	-6.41	0.494	0.088
		LPT – LPS	0.591	-2.703	3.89	0.709
Total bilirubin (µmol/L)	D1	LPS – CON	3.926	-0.539	8.391	0.081
		LPT – CON	5.445	0.760	10.130	0.025
		LPT – LPS	4.851	0.832	8.871	0.031
	D3	LPS – CON	0.626	-3.839	5.091	0.771
		LPT – CON	2.128	-2.557	6.813	0.351
		LPT – LPS	1.5016	-2.85	5.854	0.477
	D10	LPS – CON	-0.983	-5.447	3.482	0.648
		LPT – CON	-1.029	-5.715	3.656	0.649
		LPT – LPS	-0.0469	-4.40	4.305	0.982
	D30	LPS – CON	-3.264	-7.729	1.201	0.141
		LPT – CON	-5.255	-9.940	-0.570	0.030
		LPT – LPS	-1.9912	-6.34	2.361	0.348
AST (IU/L)	D1	LPS – CON	41.6	2.85	80.4	0.037
		LPT – CON	57.9	17.80	98.0	0.007
		LPT – LPS	16.25	-20.4	52.93	0.363
	D3	LPS – CON	19.3	-19.43	58.1	0.307
		LPT – CON	23.3	-16.80	63.4	0.237
		LPT – LPS	3.93	-32.7	40.61	0.824
	D10	LPS – CON	-19.6	-58.33	19.2	0.302
		LPT – CON	-24.6	-64.66	15.5	0.213
		LPT – LPS	-5.02	-41.7	31.66	0.776
	D30	LPS – CON	-56.2	-94.96	-17.4	0.007
		LPT – CON	-70.1	-110.23	-30.1	0.002
		LPT – LPS	-13.96	-50.6	22.71	0.433
GGT (IU/L)	D1	LPS – CON	6.49	0.781	12.19	0.028
		LPT – CON	5.587	0.115	11.06	0.045
		LPT – LPS	-1.012	-5.50	3.480	0.640
	D3	LPS – CON	1.77	-3.940	7.47	0.523
		LPT – CON	4.68	-0.905	10.27	0.095
		LPT – LPS	2.919	-1.57	7.411	0.188
	D10	LPS – CON	-3.12	-8.822	2.59	0.265
		LPT – CON	-2.72	-8.310	2.87	0.319
		LPT – LPS	0.396	-4.10	4.888	0.855
	D30	LPS – CON	-1.14	-6.847	4.56	0.678
		LPT – CON	-1.33	-6.920	4.26	0.622
		LPT – LPS	-0.188	-4.68	4.304	0.931
Uric acid (µmol/L)	D1	LPS – CON	79.7	4.536	155	0.039
		LPT – CON	91.1	16.603	166	0.019
		LPT – LPS	11.41	-63.0	85.78	0.750
	D3	LPS – CON	76.3	1.147	151	0.047
		LPT – CON	70.4	-4.124	145	0.063
		LPT – LPS	-5.93	-80.3	68.44	0.868
	D10	LPS – CON	68.6	-6.493	144	0.071
		LPT – CON	74.1	-0.366	149	0.051

		LPT – LPS	5.47	-68.9	79.84	0.879
	D30	LPS – CON	70.8	-4.297	146	0.063
		LPT – CON	68.4	-6.037	143	0.069
		LPT – LPS	-2.40	-76.8	71.97	0.947
DPPH (%)	D1	LPS – CON	17.1	7.13	27.0	0.002
		LPT – CON	18.5	8.04	28.9	0.002
		LPT – LPS	1.395	-8.53	11.32	0.770
	D3	LPS – CON	19.7	9.77	29.7	<0.001
		LPT – CON	18.8	8.33	29.2	0.001
		LPT – LPS	-0.955	-10.88	8.97	0.842
	D10	LPS – CON	17.8	7.82	27.7	0.002
		LPT – CON	23.7	13.25	34.1	<0.001
		LPT – LPS	5.915	-4.01	15.84	0.226
	D30	LPS – CON	21.1	11.14	31.0	<0.001
		LPT – CON	24.8	14.35	35.2	<0.001
		LPT – LPS	3.691	-6.23	13.61	0.443
PON1 (U/L)	D1	LPS – CON	-0.3033	-0.529	-0.0774	0.011
		LPT – CON	-0.3110	-0.529	-0.0930	0.008
		LPT – LPS	-0.00766	-0.2060	0.1907	0.936
	D3	LPS – CON	0.0436	-0.182	0.2696	0.689
		LPT – CON	-0.0364	-0.254	0.1816	0.729
		LPT – LPS	-0.08005	-0.2784	0.1183	0.406
	D10	LPS – CON	-0.0855	-0.311	0.1405	0.436
		LPT – CON	-0.1901	-0.408	0.0280	0.083
		LPT – LPS	-0.10461	-0.3030	0.0937	0.281
	D30	LPS – CON	-0.0579	-0.284	0.1681	0.596
		LPT – CON	-0.0940	-0.312	0.1240	0.376
		LPT – LPS	-0.03616	-0.2345	0.1622	0.705
MPO (U/L)	D1	LPS – CON	0.4705	0.2937	0.6474	<0.001
		LPT – CON	0.8848	0.7020	1.0677	<0.001
		LPT – LPS	0.4143	0.2773	0.551	<0.001
	D3	LPS – CON	-0.0768	-0.2484	0.0947	0.065
		LPT – CON	0.6172	0.4343	0.8000	<0.001
		LPT – LPS	0.8887	0.7517	1.026	<0.001
	D10	LPS – CON	-0.0247	-0.2015	0.1522	0.772
		LPT – CON	0.7070	0.5241	0.8898	<0.001
		LPT – LPS	0.7317	0.5947	0.869	<0.001
	D30	LPS – CON	-0.4508	-0.6277	-0.2740	<0.001
		LPT – CON	0.2605	0.0777	0.4434	0.008
		LPT – LPS	0.7113	0.5743	0.848	<0.001
